# Atlanta Academy of Medicine

**Published:** 1877-10

**Authors:** J. S. Todd

**Affiliations:** Reporter


### Reports of Societies.

### ATLANTA ACADEMY OF MEDICINE.

J. S. TODD, M.D., Reporter.

Atlanta, Ga., Oct. 2, 1877.

V. H. Taliaferro, M.D., President, in the Chair.

Dr. Logan spoke generally on the use of graduated short cloth tents for the cure of uterine flexions and contracted cervices. His experience, though limited, and of short character, impressed him favorably with their value as remedial agents for the above disorders. He does not regard medication of the tent as at all necessary to overcome mechanical lesion, but should there be need for intra-uterine medication, he knows of no better mode of local application to the cavity of the cervix uteri; the tent being short, just goes within the internal os.

To Dr. Taliaferro is due the credit of the introduction of the cloth tent to the profession, but, so for as he was aware, this modification and special use of this tent was original with himself.

Dr. Taliaferro asked Dr. Logan what he thought was the *modus operandi* in the cure of flexions.

Dr. Logan said he thought it acted both mechanically and as a stimulant to the parts, thereby increasing nutrition, etc. In some cases he keeps in the same tent three or four days. After removing, he allows twelve hours to elapse before inserting another. He places no restriction on exercise while they are worn. Thinks they can be worn where the sea-tangle or sponge tent cannot; in fact prefers them, and has never seen a case where they caused any local disturbance. He spoke of a case of dysmenorrhœa, dependent on contraction of the os, which was entirely relieved during the period following its application.

Dr. Armstrong had also used the tents recommended by Dr. T., with very happy results, in four cases uncomplicated with displacement. One case, that of a lady æt. 32, who had had no children for ten years. He found her bed-ridden, emaciated, etc., the uterus enlarged and congested, with catarrh; tender over the region of the ovaries. The use of the tent, medicated with iodine, for two months, resulted in the reestablishment of regular monthly periods and her entire recovery.

Dr. Taliaferro said he had relieved many bad cases of flexions with the tents. Dr. L.'s philosophy of cure he thought correct, for when the flexions resulted from fatty degeneration the stimulation afforded by the tent was highly desirable, and could not fail to do good. Dysmenorrhœa dependent on congestion was often permanently cured, but where stenosis was the cause, gratifying temporary results would ensue, but would not be permanent, for the cause—stenosis—was sure to recur in a few months.

Dr. W. F. Westmoreland to-day saw the case reported in April, of stricture with rupture of the urethra, causing infiltration, sloughing of scrotum and the tunica albuginia on one side to the crura, etc. The testes are now covered with true skin, nature having drawn it from perinenm, thighs, and the regions thereabout. The bulb of the urethra, through which he still urinates, is exposed. There has not been, since the rupture, nor can there ever again be, another erection, for the erectal tissue has been irreparably injured in the corpus cavernosa.

Dr. W. also reported two cases of cystic tumors. One occurred in an old woman, in the recto-vaginal septum. The symptoms were inability to pass urine, great difficulty in emptying bowels. Vagina and pelvis filled with the tumor. On puncturing the tumor, a quart of sanguinous fluid, clearer towards the last, and finally milky, was passed. It refilled, and has been discharged by puncture, two or three times since. The patient is too old and weak for active treatment—palliation alone being admissable. The other case a woman of thirty or forty, who is the mother of several children, has a tumor in the urethro-vaginal septum as large as a hen's egg. There were difficulty and pain in passing water, and to introduce the catheter the point had to be turned toward the symphisis pubis to enter the bladder. The variability in the size of the tumor and the absence of any connection with the bladder led him to infer (as was afterwards proved correctly) that it had a connection with the urethra, which fact was revealed by patient and diligent use of the probe. The contents were mixed pus and decomposed urine. Treatment: he extended the urethra by incisions into the cyst; true, it made a large meatus, large enough to introduce your thumb; but had he punctured through the vagina, a fistula would have been the result. The relief has been as permanent as it was sudden. The cavity, in a week or ten days has greatly contracted, and of the final favorable result he has no doubt.

Adjourned.

ATLANTA, GA., October 9, 1877.

Dr. W. F. Westmoreland reported a case of necrosis of superior and inferior maxilla, in the person of a lady, who, for seven or eight years past, has suffered greatly. He recently operated for the removal of the diseased bone. He found the necrosed bone encysted in new bone. He removed two pieces at two operations. The necrosed bone was very heavy and dense, more like stone than osseous tissue. He commenced the operation by an incision through the skin, on the left side of the inferior maxilla—an orifice only large enough to admit a surgeon's probe was found penetrating the new formation. It was through this orifice, that more or less matter had constantly discharged for years. By means of a chisel, the new bone was removed sufficiently to allow the necrosed portions to be taken away. On the opposite side he operated upon the superior maxilla through the mouth—making no external incision. The sequestra were removed by means of a strong knife and bone forceps. Has seen a great many cases of necrosis and new osseous formation, but never before met with a case like this. He proposes to make other operations at other points in the future.

Dr. Fitts, of Carrollton, who was present by invitation, presented his own case, and requested the opinion of the Academy. Dr. F. stated that, about the first of January, 1875, a hard scale appeared on the back of his right hand. He attempted to scrape it off with his knife, but the pain was such that he desisted.

It continued to grow, and in a few weeks resembled a "seed wart." He pulled out fifty or sixty small bodies from it, resembling small, hard seed—hence the name—in the course of a few months. It continued to grow until it attained a size of one and a quarter inches in diameter, and an elevation of half an inch. Last March it was cut out by his partner, but it speedily returned, and is now as large as ever. It presents a small abraded point on the surface, from which pus and sebaceous matter is occasionally discharged.

It is excessively painful. The pain is sharp and lancinating. He has not slept all night for twenty months, on account of it, except for a few days after its removal last spring, when it was free from pain. What is it, and what course of treatment should be pursued?

Dr. W. F. Westmoreland arrived at the conclusion that the growth was epithelioma. He was impressed with this idea from the character of the first growth, and its recurrence after removal It does not now present all the characteristics of epithelioma, but it will not be long before the history is complete. It will become malignant, and if not removed will cost the Doctor his life. It should be done thoroughly. It was not completely removed at the first operation.

For this case prefers caustic. It is a slow process, more tedious, but more effectual, than the knife, in that locality. It is impossible to say to what extent the surrounding tissue is implicated. Recommended the anhidrous sulphate of zinc. Has observed that it destroys diseased tissue with little effect upon the surrounding healthy structure. He applies a paste with glycerine as long as it will cause a slough.

Has recently treated fifteen or twenty cases by this means, in situations where the knife would have been inadmissible, with complete success. A remarkable fact in connection with this mode of treatment, is the very small scar that remains after healing. He accounted for its discrimination between sound and diseased tissues, on the ground that the latter were of lower vitality and do not so effectually resist the action of the remedy. When this caustic is used, it is frequently omitted too soon. Its application should be repeated again and again, if necessary, until it fails to produce a slough. This is an important point to bear in mind. Its application is frequently attended with intense pain for an hour or two. This may be prevented, or greatly lessened, by the local use of carbolic acid, the anæsthetic properties of which were first brought to his attention by Dr. Doughty, of Augusta.

The acid itself, if applied pure to the skin, will produce great pain. His plan is to commence by using one part of the acid to twenty of water, then half and half, and as the sensibility is thus gradually benumbed, the full strength, when it is required, may be safely employed, and, finally the caustic may be applied without fear of causing excessive suffering, and, in many cases, no pain whatever. It is necessary that the surface should be abraded, or scarified, before the caustic will act.

Dr. Logan alluded to Sir James Y. Simpson's praise of the anhydrous sulphate of zinc as a caustic, and his positive assertion that it would always discriminate between the healthy and diseased parts. It has yielded, always, splendid success in his hands. He referred to a case of epithelioma of the nose treated by this means, about eighteen months ago, with satisfactory results.

Dr. Jas. F. Alexander spoke of its action on a warty excrescence upon the nose, under his observation. The paste was applied upon a piece of adhesive plaster. It was allowed to remain for two or three days. It was then removed, and the ulcer which remained speedily healed, and left no perceptible cicatrix.

Adjourned.

---

### ACTION OF THE ATLANTA ACADEMY OF MEDICINE UPON THE DEATH OF DR JAMES F. BOZEMAN.

ATLANTA, GA., Oct. 16, 1877.

Academy met. President, Dr. V. H. Taliaferro, in the Chair.

The Committee appointed to draft resolutions expressive of the feelings of members on the death of Dr. Bozeman reported as follows:

Your Committee respectfully submit the following report:

It is with deep regret that we record the death of one of our honorary members, Dr. James Fort Bozeman, of this city, which sad event occurred on the 10th of September last, after a very brief illness. The deceased was born May 16, 1824, in the city of Milledgeville, Ga., received his collegiate education at Oglethorpe University, and graduated from the Medical Department of the University of Pennsylvania, in 1846. The following year he commenced the practice of his profession, at Columbus, Ga., but, in 1849, was appointed Assistant Surgeon in the United States army, and was ordered to duty in Mexico, with the Georgia Mounted Volunteers. At the close of the Mexican war, Dr. Bozeman returned to Columbus, resumed his private practice, and continued it until 1862, when ill health compelled him to retire from the duties of his profession. In 1866 they were, however, resumed, but again abandoned, for the same reason, in a few months. He removed to Atlanta, in 1871, and again returned to his profession, but after a year, owing to feeble health, it was permanently abandoned.

From that period to the time of his death, Dr. Bozeman was engaged in financial operations.

For several years previous to this he was President of the Georgia Home Insurance Company, of Columbus, of which city he was at one time Mayor.

He was, at the time of his death, President of the Board of Trustees of the State Insane Asylum, Director of the Central and other railroads, and Financial Agent of the State of Georgia.

Varied as were his professional and public services, the deceased has left behind him a record for faithfulness in the discharge of duty that should commend itself to all who desire to attain and deserve positions of honor and usefulness.

Dr. Bozeman held no public or private trust, assumed no obligation during his long and active career that was ever betrayed, or discharged without credit to himself. His last public service was an able and unanswerable defense of his native State from the base charge of having repudiated a portion of her just obligations.

In view, therefore, of his private worth, professional acquirements and public services, it is hereby—

*Resolved*, That, in the death of Dr. Jas. F. Bozeman, society, the church, the medical profession, and the State, have lost a valued, an honored and eminently useful man, whose private and public career is worthy of emulation.

*Resolved*, That the Atlanta Academy of Medicine, of which the deceased was an honorary member, while deploring its own loss, extend to the bereaved family the most sincere sympathy in their deep and sudden affliction.

J. P. Logan, M.D.,
W. S. Armstrong, M.D.,
J. S. Todd, M.D.,
*Committee.*

The report was unanimously adopted, and, on motion, was ordered spread upon the minutes of the Academy, and the Secretary was instructed to send a copy to the family of the deceased, and to furnish a copy each to the Atlanta Medical

AND SURGICAL JOURNAL and the Atlanta *Constitution* for publication.

V. H. TALIAFERRO, M.D., *President.*

JAS. B. BAIRD, M.D., *Secretary.*

